# IgG4-related disease with tracheobronchial miliary nodules and asthma: a case report and review of the literature

**DOI:** 10.1186/s12890-019-0957-9

**Published:** 2019-10-30

**Authors:** Xiuling Wang, Jun Wan, Ling Zhao, Jiping Da, Bin Cao, Zhenguo Zhai

**Affiliations:** 1 Center for Respiratory Diseases, China-Japan Friendship Hospital; Department of Pulmonary and Critical Care Medicine, China-Japan Friendship Hospital; National Clinical Research Center for Respiratory Diseases, Peking University Health Science Center, No 2, East Yinghua Road, Chaoyang District, Beijing, 100029 People’s Republic of China; 2 Fuwai Hospital, National Center for Cardiovascular Diseases, Chinese Academy of Medical Sciences and Peking Union Medical College, No 167, Beilishi Road, Xicheng District, Beijing, 100037 People’s Republic of China; 3 Department of Pathology, China-Japan Friendship Hospital, No 2, East Yinghua Road, Chaoyang District, Beijing, 100029 People’s Republic of China

**Keywords:** IgG4-related disease, Pulmonary involvement, Tracheobronchial nodules

## Abstract

**Background:**

IgG4-related disease (IgG4-RD) is a systemic autoimmune disease that can affect multiple organs of the body. Pulmonary manifestations of IgG4-RD include pulmonary solid nodules, thickening of bronchovascular bundles, interstitial involvement, and ground glass opacities. Here we present a rare case of IgG4-RD with tracheobronchial nodules and review the relevant literature.

**Case presentation:**

A 52-year-old man was admitted to our hospital with a history of intermittent cough for 27 months and recurrent wheezing for 17 months. He had been diagnosed with asthma prior to admission and was responsive to oral prednisone (30 mg/day, with gradual tapering). Bronchoscopy performed 2 years prior to admission showed tracheal and bronchial mucosal hyperemia, edema, and miliary nodules. Pathological tests showed chronic inflammation with focal lymphocytic infiltration in the bronchial mucosa. The patient had recurrent cough and wheezing after prednisone was stopped or the dose reduced. At the time of admission to our hospital, his serum immunoglobulin G4 (IgG4) level had increased to 7.35 g/L. Following bronchoscopy, the IgG4 expression in the bronchial mucosa was compared with that observed during the last two bronchoscopies. Bronchoscopy performed 7 months prior to admission revealed IgG4+ plasma cell infiltration in the bronchial tissue, with > 10 IgG4+ plasma cells per high power field and an IgG4+/IgG+ cell ratio of > 40%. The current bronchoscopy revealed a decrease in IgG4 expression in the bronchial tissue, probably because of the intermittent prednisone treatment. The case fulfilled the comprehensive clinical diagnostic criteria for IgG4-RD. He received prednisone and azathioprine, and he has never developed recurrence.

**Conclusions:**

Our case exhibited three important clinical indication: First, tracheobronchial miliary nodules could be the presentation of IgG4-related disease. Second, IgG4-related disease with pulmonary involvement has close connection with asthma. Last, IgG4-related disease can be very sensitive to prednisone, the infiltration of IgG4 positive plasma cells decreased after prednisone treatment and symptoms significantly improved in our case. In conclusion, we reported the first case of IgG4-RD presenting with miliary nodules on the tracheal and bronchial tube walls combined with asthma. The findings will further our understanding of the characteristics of IgG4-RD.

## Background

IgG4-related disease (IgG4-RD) is a systemic autoimmune disease first described by Hamano H et al. in 2001 [1]. It is characterized by infiltration of immunoglobulin G4 (IgG4)-bearing lymphoplasmacytic cells in the involved organs, which include the pancreas, biliary tree and gallbladder, major salivary glands, ocular region, lung, and kidneys. Pulmonary manifestations include pulmonary solid nodules, thickening of bronchovascular bundles and interlobular septa, interstitial involvement, and round-shaped ground glass opacities (Table 1). Here we report a pathologically confirmed case of IgG4-RD with tracheobronchial nodules.

**Table 1:** Usual imaging manifestations of pulmonary involvement of IgG4-RD

Location	Imaging Manifestations
Pulmonary parenchyma [22]	Ground glass opacities
	Solid nodules/masses
	Interstitial lung disease
Airway/Vasculature [23]	Enlargement of bronchovascular bundle
Mediastinum	Enlargement of lymph node [14]
	Mediastinal mass [24]
Pleural	Pleural thickening [25]
	Pleural mass [25]
	Pleural effusion [26]

## Case presentation

**Fig. 1:**
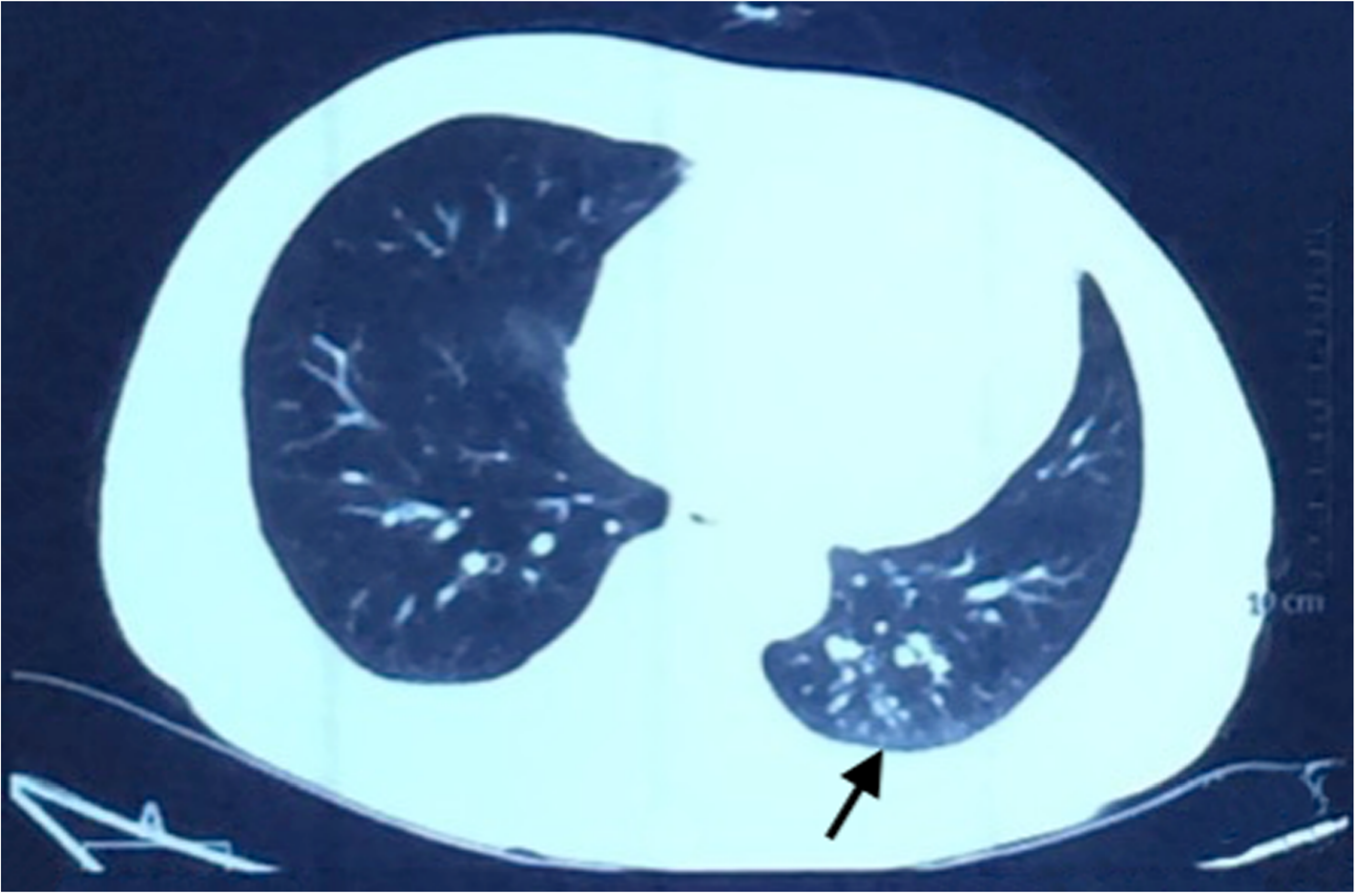
Lung CT scanning (2014-09-15). Lung CT scanning showed infiltration of left lower lung indicated by an arrow, informing left lower lobe pneumonia

**Fig. 2:**
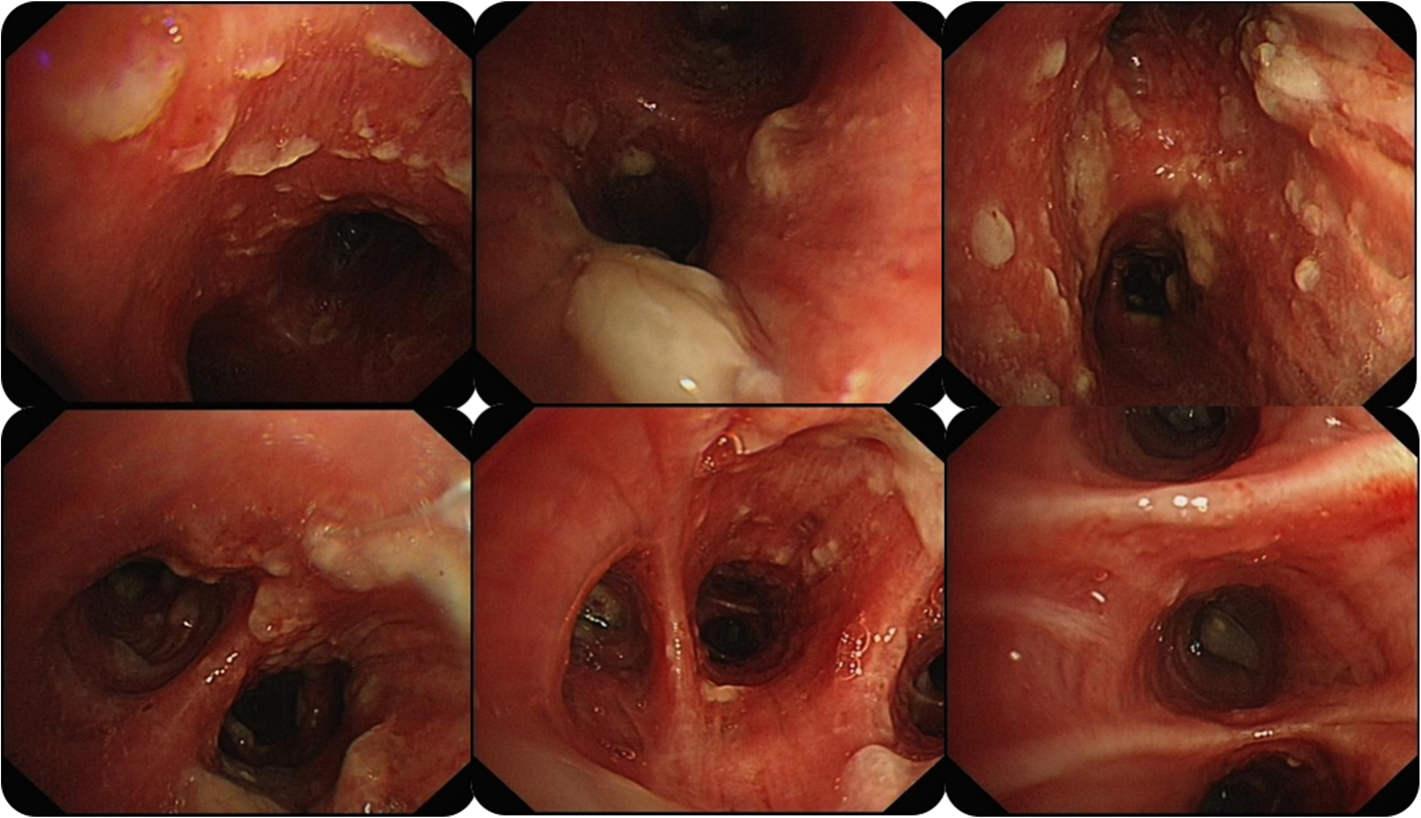
Bronchoscopy findings (first time). The bronchoscopy showed multiple white nodular protuberance in trachea and bronchus, along with mucosal hyperemia and edema

**Fig. 3:**
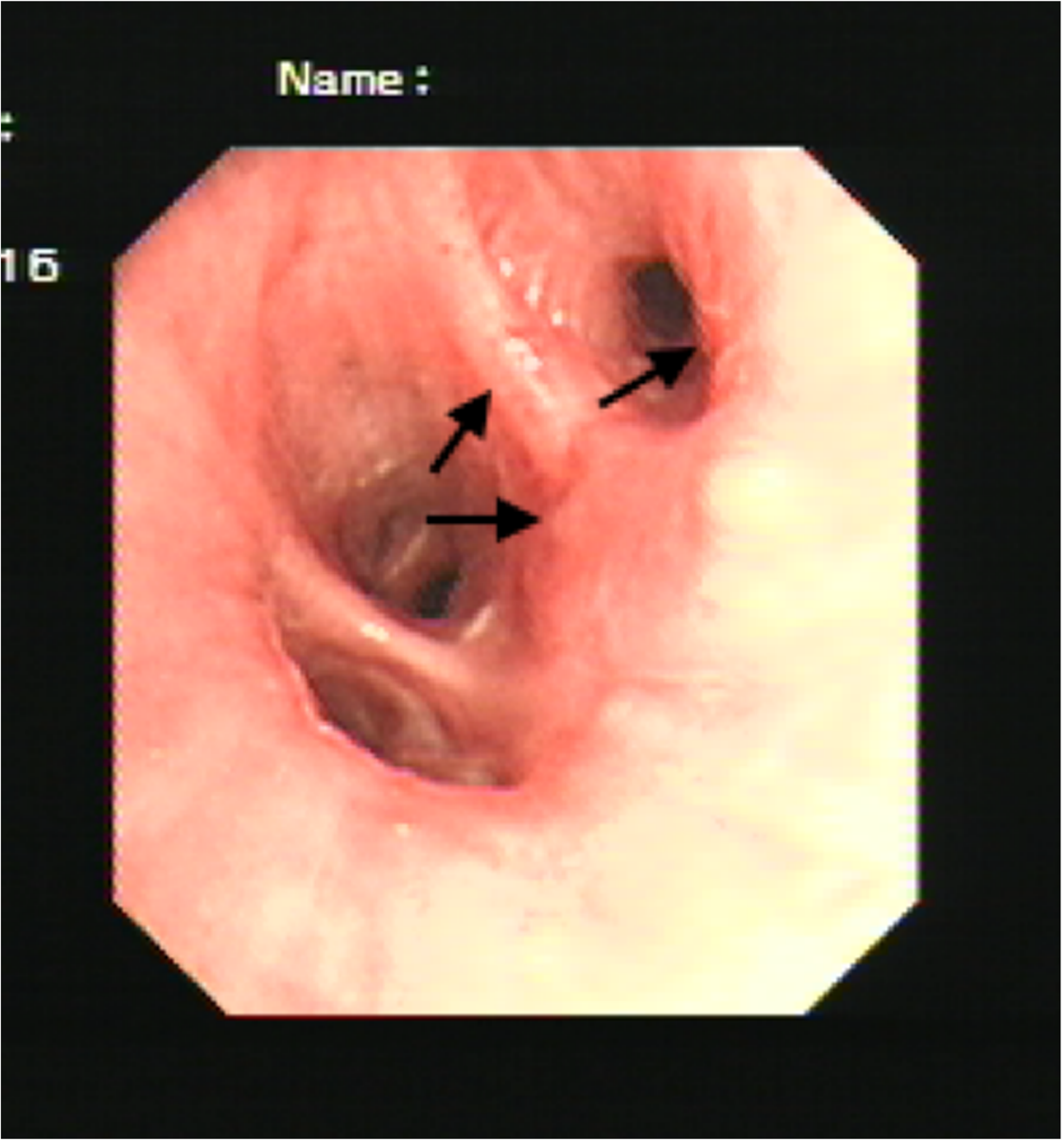
Bronchoscopy findings (second time). The alleviated miliary nodules on the trachea and bronchus tube wall indicated by arrows

**Fig. 4:**
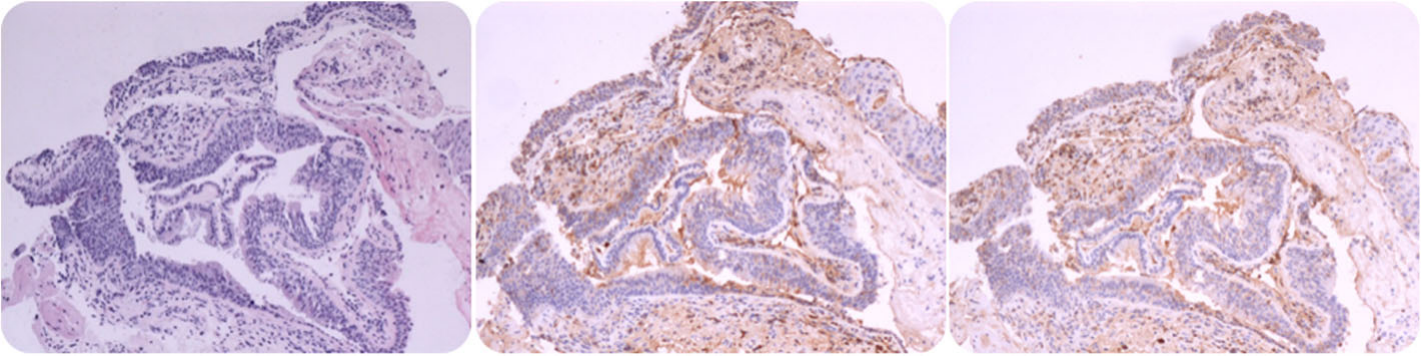
Tracheal mucosa pathology (first time). Infiltration of IgG4+ plasma cells, the ratio of IgG4+/IgG+ cells> 40%, and IgG4+ plasma cells> 10/HPF, figures are as follows separately (from left to right):HE stain magnification 4 times; IgG stain magnification 10 times; IgG4 stain magnification 10

**Fig. 5:**
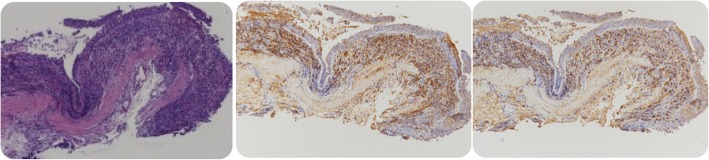
Tracheal mucosa pathology (second time). The expression of IgG4 decreased in the tissue of 2016. Figures are as follows separately (from left to right):HE stain magnification 10 times; IgG stain magnification 10 times; IgG4 stain magnification 10 times

A 52-year-old man was admitted to our hospital with a history of intermittent cough for 27 months and repeated wheezing for 17 months. The patient developed cough and cold 27 months back. The cough aggravated at night and was accompanied by snuffling and a sore throat. After treatment with antibiotics, all symptoms but cough improved, and the cough worsened over time. In the last 17 months, the patient also complained of wheezing accompanied by jelly-like sputum, usually after exposure to cold air. He was then admitted to a local hospital. Lung computed tomography (CT) performed at the local hospital showed infiltration in the left lower lung (Fig. 1), indicating left lower lobe pneumonia. A complete blood count yielded the following results: white blood cells, 5.5 × 109/L; neutrophils, 46.7%, and eosinophils, 5.3%. Pulmonary function tests showed normal ventilation function. Bronchoscopy revealed tracheal and bronchial mucosal hyperemia and edema and miliary nodules. Histopathology revealed chronic inflammation of the bronchial mucosa with focal lymphocytic infiltration. The patient was treated with antibiotics, expectorants, and bronchodilators; however, his cough symptoms showed no significant improvement. After 1 month, he caught a cold again and developed cough and wheezing. He was then diagnosed with asthma and treated with prednisone (30 mg/day orally, with gradual tapering) for 1.5 months. His coughing and wheezing symptoms ameliorated, although they recurred when prednisone was stopped. Bronchoscopy performed 7 months prior to admission (Fig. 2) showed multiple white nodular protuberances in the trachea and bronchus, with mucosal hyperemia and edema. Furthermore, mucosal pathology showed chronic inflammation with focal squamous metaplasia. A large number of eosinophils were found in the bronchoalveolar lavage fluid smear. Prednisone (30 mg/day) was restarted, but his condition relapsed when the dose was tapered to 5 mg/day. The proportion of eosinophils in the peripheral blood increased to 31% (absolute blood eosinophil count: 3.21 × 10^9^/l), with a serum IgE level of 4220 IU/ml. On admission to our hospital, his routine physical examination findings were unremarkable. His serum IgG4 level had increased to 7.35 g/L. Lung CT showed no abnormality. The pulmonary function test results were as follows: TLC %: 99.2%, RV %: 96.6%, RV/TLC: 93%, FVC%: 77.8%, VT%:154.1%, FEV1/FVC: 88.71%, FEV1%: 83.8%, DLCO-SB%: 67.8%, DLCO/VA:100.3%, and the bronchial dilation test was positive. His symptoms and pulmonary function test results were consistent with the diagnosis of asthma. However, the inhalation treatment (Symbicort turbuhaler, 160/4.5 micrograms/inhalation, 1 inhalation twice a day) cannot improve his symptoms. We give him a repeated bronchoscopy which suggested scattered alleviated nodules under the tracheobronchial wall without obvious mucosal hyperemia and edema, which improved a lot compared with the bronchoscopy 7 months ago. (Fig. 3). Considering that he is very sensitive to the prednisone, the tissue biopsy obtained at our hospital was stained to identify IgG4 expression and revealed low expression of IgG4 (Fig. 4), with the presence of phlebitis and fibrosis. We detected IgG4 expression in the mucosal tissues from the bronchoscopy performed 7 months earlier. The biopsy showed chronic inflammation with significantly increased plasm cell infiltration and IgG4+ plasma cell infiltration, along with elevated eosinophils. The IgG4+/IgG+ cell ratio was over 40%, and there were more than 10 IgG4+ plasma cells per high power field (HPF) (Fig. 5). The biopsy excluded malignancy and other diseases involving IgG4+ plasma cell infiltration, including sarcoidosis, Wegener’s granulomatosis, Churg–Strauss syndrome, and multicentric Castleman’s disease. Other laboratory tests of the patient including anti-PR3, anti-MPO ANCA, myositis specific antibody, Anti-nuclear Antibody, ACE, serum G-test, serum GM-test, bacterial and fungal culture of bronchoalveolar lavage fluid (BALF) were all normal. We excluded other organ involvement, including pancreas dysfunction and retroperitoneal fibrosis. As recommend by the guideline [2], the patient was diagnosed with IgG4-RD and prednisone treatment was continued (24 mg/day, orally, gradually tapered). He took cyclophosphamide (100 mg/day) while the prednisone dose was gradually tapered. Currently, he is receiving prednisone 12 mg and azathioprine 100 mg/day with good drug adherence by self-reported, and he has not developed recurrence after hospital discharge. Six months later, his serum IgG4 level and blood eosinophil count decreased to the normal level (serum IgG4: 164 mg/d, blood eosinophil count: 0.12*10^9/l). A repeat low dose lung CT scan didn’t find new lesions.

## Discussion and conclusions

IgG4-related disease (IgG4-RD) is an emerging immune-mediated disease that can affect any body organ. In 1961, Sarles et al. [2] first reported a case of pancreatitis with hypergammaglobulinemia, suggestive of an underlying autoimmune process. In 2001, Hamano et al. [1] first established the association between sclerosing pancreatitis and elevated serum concentrations of IgG4. Subsequently, in 2003, Kamisawa et al. [3] found that this autoimmune process is not limited to the pancreas and is actually a systemic disease. The authors introduced the term “IgG4-related autoimmune disease”. The term IgG4-RD was finally proposed in 2012 by an international multidisciplinary study group, and a consensus was reached about its pathology [4]. The first management and treatment guidelines for IgG4-RG were published in 2015 [5].

The widespread incomplete understanding of IgG4-RD has led to misdiagnosis of the disease. Because IgG4-RD can be controlled well by glucocorticoids, it is important to study its characteristics for correct diagnosis. In 2012, IgG4-RD study groups in Japan published the first comprehensive clinical diagnostic criteria [6]. The criteria emphasize diffuse/localized swellings or masses in single or multiple organs and elevated serum IgG4 concentrations (135 mg/dl), with marked lymphocyte and plasmacyte infiltration and fibrosis, infiltration of IgG4+ plasma cells, an IgG4+/IgG+ cell ratio of > 40%, and > 10 IgG4+ plasma cells/HPF in histopathological examination. In 2015, an international panel of experts [5] further pointed out that the diagnosis of IgG4-RD should be based on comprehensive criteria, including a full clinical history, physical examination, selected laboratory investigations, and appropriate radiological studies. Furthermore, biopsy is strongly recommended for the exclusion of malignancies and other IgG4-RD mimics. In this case report, the serum IgG4 level was substantially increased, and the mucosal biopsy specimen showed infiltration of IgG4+ plasma cells, an IgG4+/IgG+ cell ratio of > 40%, and > 10 IgG4+ plasma cells/HPF. The patient was responsive to glucocorticoid treatment. Furthermore, his symptoms, physical examination, laboratory tests, and biopsy excluded malignancy and other diseases involving IgG4+ plasma cell infiltration, including sarcoidosis, Wegener’s granulomatosis, Churg–Strauss syndrome, and multicentric Castleman’s disease. Therefore, the patient’s diagnosis of IgG4-RD was confirmed by comprehensive clinical information.

To our knowledge, this is the first reported case of IgG4-RD presenting with miliary nodules on the tracheal and bronchial tube walls, which is a rare type of IgG4-RD. Two other cases of IgG4-RD with isolated tracheobronchial involvement presenting as mass-like lesions have been reported [7, 8], in addition to five cases [9–13] of systemic IgG4-RD with tracheobronchial involvement and tracheobronchial edema or capillary dilatation. We found no difference in pathology between the cases of systemic IgG4-RD and those of isolated tracheobronchial involvement.

Our patient was diagnosed with asthma; he had a positive bronchodilator reversibility test. He suffered from recurrent cough and wheezing that aggravated at night and on exposure to cold air. Furthermore, he had increased eosinophils in the peripheral blood and bronchoalveolar lavage fluid smear. The asthma diagnosis was in line with previous studies reporting patients with a diagnosis of IgG4-RD and asthma [8, 10] (Table 2, cases 2 and 6, respectively). Indeed, several studies and case reports have found connections between IgG4-RD and asthma. A cross-sectional study [14] found that 12% IgG4-RD patients had been diagnosed with asthma. Furthermore, Flament et al. [15] found that several patients with asthma patients had elevated serum IgG4 concentrations. These patients had significantly high blood eosinophilia, total IgE concentration, and fractional exhaled nitric oxide value.

**Table 2:** Seven cases of IgG4 related Tracheobronchial lesions

Case Age/sex	Respiratory symptom	Tracheobronchial lesions feature	Serum IgG4 (mg/dl)	Extra-airway involvement	Treatment	Pathology
1 [11] 63/f	Cough	Mucosal edema and engorged vessels	1660	Submandibular gland neoplasm, autoimmune pancreatitis	oral prednisolone 1 mg*kg^−1^*d^−1^. All involvement improved	biopsy specimens from the bile duct showed infiltration of IgG4-positive plasma cells. Bronchial biopsy:diffuse inflammatory infiltrates consisting mainly of plasma cells, lymphocytes and scattered eosinophils with fibrosis with infiltration of several IgG4-positive plasma cells. The number of IgG4- positive cells was 30 per HPF。
2 [8] 22/f	Shortness of breath, wheezing, sore throat	mass surrounding larynx and upper trachea	N/A	none	Prednisolone and surgery	an IgG4-sclerosing pseudotumor, with fibrosis and a dense acute-on- chronic inflammatory infiltrate rich in plasma cells. This was associated with a proliferation of histiocytes and aggregates of lymphocytes. Immune-staining demonstrated mixed CD20+ B lymphocytes and CD3+ T lymphocytes. CD68 elucidated scattered histiocytes. The IgG/IgG4 plasma cell ratio was less than 50%.
3 [12] 70/M	None	Edematous and multiple central lesions and capillary dilatation in the primary bronchi	2600	Submaxillary gland and Parotid gland swelling, hypertrophic pachymeningitis	oral prednisolone	A lumbar puncture revealed pleocytosis (29.6/mm^3^: mononuclear,25.6/mm^3^, polymorphonuclear, 4/mm^3^) Biopsy specimens of the parotid gland and a bronchial elevated lesion: chronic inflammation and fibrosis in both lesions, as well as numerous plasma cell infiltrations. Immunohistochemical analysis indicated that the majority of plasma cells were IgG4+.
4 [7] 26/F	Shortness of breath and persistent dry cough	A tracheal lesion with 90% luminal obstruction	N/A	None	surgery	a dense lymphoplasmacytic infiltrate and fibrosis in a storiform pattern. The infiltrate was composed predominantly of lymphocytes and plasma cells, with interspersed fibroblasts and eosinophils. Immunostaining showed abundant IgG4-positive cells (155 per high-power field) and an IgG4/IgG ratio of approximately 0.9.
5 [9] 44/M	Sinus congestion, wheezing, dyspnea and cough	Inflammatory changes along the tracheobronchial tree.	2020	Pulmonary Parenchymal infiltrates, intrathoracic lymphadenopathy, submandibular gland swelling and a kidney mass.	oral prednisone with 7.5 mg of maintenance therapy	Immunostaining for IgG4 highlighted 15 to 20 IgG4-positive plasma cells per high-power field. Outside submandibular gland pathology demonstrated numerous IgG4-positive plasma cells with an IgG4/IgG cell ratio of 0.6. Submandibular gland biopsy demonstrating the features of chronic sialadenitis. Increased immunoglobulin IgG4-positive plasma cells within the chronic inflammatory infiltrate in submandibular gland biopsy. Bronchoscopic biopsy demonstrating chronic inflammatory infiltrate and thickened basement membrane in bronchial mucosa. Increased IgG4-positive plasma cells within the inflammatory infiltrate in bronchial mucosa.
6 [10] 70/F	Dyspnea and facial edema	A smooth polypoid mass at the lower trachea.	N/A	Mass in the superior vena cava. 15 years ago: a mediastinal mass in the intratracheal and right lower paratracheal area	surgery	a mediastinal mass 15 years ago: diffusely fibrosclerotic change with proliferation of the fibroblasts and infiltration of chronic inflammatory cells. mass in the superior vena cava:markedly increased lymphoid follicles, fibrosclerotic change of the stroma and a heavy infiltration of the plasma cells. In addition, immunohistochemical staining for IgG4 antibody demonstrated diffusion with strong positivity at the increased plasma cells
7 [13] 50/M	Chronic cough	Marked edema of the bronchial mucosa.	1180	Autoimmune pancreatitis, sinus mucosa thickening	Inhaled corticosteroids with systemic corticosteroid therapy	A bronchial biopsy specimen showed inflammation with marked infiltration of IgG4-positive plasma cells and storiform fibrosis.

*F* Female, *M* Male, *N/A* Not available, *HPF* High-power field

The increased level of serum IgE in our patient was suggestive of an allergic immunological response in vivo. A previous study found that 44% patients with autoimmune pancreatitis had allergic diseases [16]. Similarly, other studies found that IgG4-RD and allergic diseases share a common immune characteristic, i.e., the predominance of Th2 cytokines. These can produce the Th2-related cytokine interleukin (IL)-10, which is related to the production of IgE and IgG4 [17–19]. Jeannin et al. found that Th2-related cytokines could induce the switch from IgE to IgG4 [20]. Other studies proposed that IgG4 can act as a blocking antibody against IgE-mediated allergic responses [21]. However, there is limited evidence to support any relationship between the onset and severity of allergic disease and IgG4-RD. Future studies are required for understanding the pathogenesis of allergic diseases and IgG4-RD and the relationship between them.

IgG4-RD is a newly recognized systemic autoimmune disease. Our case exhibited three important clinical indication: First, tracheobronchial miliary nodules could be the presentation of IgG4-related disease. Second, IgG4-related disease with pulmonary involvement has close connection with asthma. Last, IgG4-related disease can be very sensitive to prednisone, the infiltration of IgG4 positive cells decreased after prednisone treatment and symptoms significantly improved in our case. In conclusion, we reported the first case of IgG4-RD presenting with miliary nodules on the tracheal and bronchial tube walls combined with the diagnosis of asthma. The findings from this case may advance our understanding of IgG4-RD and contribute to its diagnosis. Future studies are warranted to aid in early diagnosis and development of suitable therapies. Besides, the relationship between IgG4-related disease and asthma need further exploration.

## Data Availability

The datasets used and/or analyzed during the current study are available from the corresponding author on reasonable request.

## Abbreviations

CT: Computed tomography
HPF: High power field
IgG4-RD: IgG4-related disease
